# Dynamic mechanical stimulation of alveolar epithelial-fibroblast models using the Flexcell tension system to study of lung disease mechanisms

**DOI:** 10.3389/fmed.2025.1552803

**Published:** 2025-08-18

**Authors:** Safiya Al Yazeedi, Tony Ju Feng Guo, Joban Sohd, Filsan Ahmed Abokor, Janaeya Zuri Baher, Logan Yee, Chung Cheung, Don D. Sin, Emmanuel Twumasi Osei

**Affiliations:** ^1^Department of Biology, University of British Columbia - Okanagan Campus, Kelowna, BC, Canada; ^2^Centre for Heart Lung Innovation, St. Paul's Hospital, Vancouver, BC, Canada

**Keywords:** *in vitro* models, mechanical model, alveolar 3D epithelial-fibroblast model, Flexcell, multicellular 3D co-culture and organoid models

## Abstract

Mechanical strain plays a significant role in lung pathophysiology. Current two-dimensional (2D) *in vitro* models fail to capture the lung's dynamic mechanical environment. We developed mechanically strained 2D and more complex three-dimensional (3D) alveolar epithelial-fibroblast co-cultures and organoids using the Flexcell cell stretching bioreactor. To do this we used readily available human A549 epithelial cells and MRC-5 lung fibroblasts to establish 2D and 3D alveolar co-cultures and collagen-I-gel-embedded organoid models in the Flexcell and then strained them at 18% amplitude, 0.4 Hz for 24 h to mimic a pathological environment. The impact of mechanical strain on cell proliferation, morphology, cytoskeletal and tight junctional protein expression, interleukin-6 and-8 (IL-6, IL-8) inflammatory cytokine release, and cell death were assessed. Mechanical strain significantly increased total cell counts in 3D co-cultures but not in 2D co-cultures, potentially signifying increased proliferation. Morphological analysis revealed a marked transition of fibroblasts into broadened shape cells under strain in the 3D co-cultures. This was in line with increased F-actin in 3D co-cultures after strain. The tight junctional protein zonula occludens-1 expression decreased after strain in all 2D and 3D models. Furthermore, exposure to strain increased the release of IL-6 and IL-8. Strain-induced cell death was also elevated across all models, particularly in 3D cultures. This study presents exploratory findings suggesting that *in vitro* mechanical multicellular alveolar models using the Flexcell system may replicate the dynamic environment of *in vivo* lung tissue. These multicellular models offer a valuable platform for investigating strain-induced cellular responses relevant to inflammatory and fibrotic mechanisms in lung diseases, particularly in exploring epithelial–mesenchymal interactions that may underlie disease progression.

## Introduction

During each breathing cycle, the lungs rhythmically expand and contract, exposing tissues to distinct mechanical forces, including compression, shear stress, and cyclic uniaxial or equibiaxial strain. These forces vary by lung region and structure and are essential for respiration, tissue homeostasis, cellular signaling, and overall lung function (1–3). In line with this, the lungs experience ~10^9^ strain cycles due to tidal breathing over a lifetime. Tidal volumes are ~5–7 mL/kg of predicted body weight, or 500 mL for a 70-kg adult [(4), p. 05] and functional residual capacity around 3.5 L (5), which corresponds to a volume strain of ~14% (6). Assuming isotropic volume expansion, this results in a uniaxial strain, or the length change of a structure per unit initial length, of 4%. In contrast, during sighs or deep inspirations, volume changes are larger, inducing linear strains of up to ~25% (1). Measurements near total lung capacity show epithelial surface area increases of around 27–37%, consistent with substantial stretching at the alveolar level (1, 7). These cyclic mechanical forces are critical for maintaining alveolar function (8).

The alveoli are primarily composed of alveolar type I (ATI) and type II (ATII) epithelial cells, apposed to underlying fibroblasts in the alveolar walls (9, 10). These cells form the barrier across which gas exchange occurs and contributes to, the maintenance of structural integrity, immune functions, and response to the lung's cyclic strain (11–13). ATI cells, which cover the majority of the alveolar surface, stretch and compress while maintaining the delicate blood-gas barrier required for gas exchange (14). ATII cells secrete surfactant to reduce alveolar surface tension and prevents atelectasis, together with their function as stem cells during injury (15). Lung fibroblasts on the other hand produce extracellular matrix (ECM) components such as collagen and elastin, providing structural support and enabling lung elasticity (16, 17).

Mechanical forces can directly influence surfactant production in ATII cells, particularly during deep breathing or strenuous activity (18). Similarly, fibroblasts respond to mechanical forces by modulating their activity, including the production and remodeling of ECM proteins such as collagen and elastin (19). Under normal conditions, this repair process is crucial for preserving the lung tissue's elastic properties, which are essential for normal lung function (11). The process by which alveolar epithelium and fibroblasts respond to mechanical changes in their physical environment via the conversion of mechanical stimuli into biochemical signals is known as mechanotransduction (20). This mechanism is critical for maintaining tissue homeostasis and plays a key role in the pathogenesis of lung diseases such as asthma, chronic obstructive pulmonary disease, idiopathic pulmonary fibrosis, and ventilator-induced lung injury (21–24).

Previous studies using the Flexcell system—a computer-controlled bioreactor designed to apply static or cyclic uni- or equibiaxial strain to various cell types—have demonstrated, through monoculture models that pathological mechanical strain disrupts cytoskeletal reorganization in alveolar epithelial cells, reduces cell viability via apoptosis and necrosis, and amplifies pro-inflammatory cytokine production (25–29). In separate studies with lung fibroblast models, pathological strain also alters proliferation, apoptosis, ECM remodeling, and production of pro-inflammatory mediators while driving chronic inflammation and fibrosis (30–33).

Although traditional two-dimensional (2D) monoculture (mechanical) models have yielded valuable insights, they fail to capture the multicellularity and three-dimensional (3D) complexity of the *in vivo* lung environment (34). This limitation reduces their accuracy in modeling disease mechanisms and potentially investigating epithelial-mesenchymal crosstalk (35). To address these limitations, the current study presents a brief research report where three *in vitro* multicellular alveolar models were established as a proof-of-concept in the Flexcell including, (i) 2D epithelial-fibroblast alveolar co-cultures, valued for their simplicity and ease of manipulation (36); (ii) 3D fibroblast-embedded collagen gels with an overlying alveolar epithelial layer; and (iii) alveolar epithelial-fibroblast organoids; both of which more closely replicate native lung tissue architecture and physiological relevance (37). These *in vitro* models were subjected to cyclic equibiaxial strain using the Flexcell system, enabling precise control over strain amplitude-waveforms and frequencies to simulate pathological conditions. In line with cellular endpoints determined from previous mechanical strain data (11), indices such as cellular proliferation, morphological changes, cell death, and inflammatory mediator release were then assessed after applying strain to characterize the multicellular alveolar models. This brief research report is the first to characterize the physiological responses of multiple relevant multicellular alveolar models to pathological strain within a single study. It establishes a foundation for future research into the mechanisms underlying lung pathologies associated with impaired mechanical environments.

## Methods

### Cell culture

A549 (CCL-185) immortalized human alveolar epithelial cells and MRC-5 (CCL-171) immortalized human lung fibroblasts were both sourced from the American Type Culture Collection (ATCC), (Manassas, Virginia, USA). Both cell-lines were grown in Dulbecco's Modified Eagle Medium (DMEM), (Thermo Fisher Scientific, Waltham, Massachusetts, USA) enriched with 1% penicillin-streptomycin (P/S), (Thermo Fisher Scientific) and 10% fetal bovine serum (FBS), (Thermo Fisher Scientific) (DMEM 10% FBS), which was replaced every 48 h. All A549 and MRC-5 cell cultures were maintained under standard conditions in a humidified incubator at 37°C with 5% CO_2_. When the cells reached 90% confluence, they were subcultured with 0.25% Trypsin-EDTA (Thermo Fisher Scientific). A549 cells were used for experiments up to passage 25 while MRC-5 fibroblasts were used till passage 16 to maintain cellular integrity. Counting and viability assessment was done using the Countess™ 3 automated cell counter (Thermo Fisher Scientific) according to the manufacturer's instructions in combination with the trypan blue exclusion method.

### Establishing 2D alveolar epithelial-fibroblast monoculture and co-culture models

Collagen-I-coated BioFlex^®^ 6-well flexible-bottom culture plates (Flexcell International Corporation, Burlington, NC) were used to create 2D alveolar epithelial and lung fibroblast monoculture as well as co-culture models. For monocultures, wells in 6-well collagen-I-coated BioFlex^®^ plates were seeded with either 100,000 MRC-5 lung fibroblasts or A549 epithelial cells and allowed to attach on the flexible collagen-I-coated BioFlex membrane for 24 h under standard conditions (37°C, 5% CO_2_ in a humidified incubator). After this initial culture period, the medium was replaced with fresh DMEM containing 1% FBS and 1% P/S. For the 2D co-cultures, 50,000 A549 epithelial cells and 50,000 MRC-5 fibroblasts were co-seeded in collagen-I-coated 6-well BioFlex^®^ plates. Co-cultures were cultured and allowed to adhere to the flexible collagen-I-coated membrane for 24 h in DMEM 10% FBS (Thermo Fisher Scientific) before replacing the medium with DMEM 1% FBS for experiments.

### Establishing 3D alveolar epithelial-fibroblast co-culture and organoid models

3D co-culture models were created in the 6 well Tissue Train circular foam culture plates (Flexcell International Corporation) by resuspending 100,000 MRC-5 lung fibroblasts in 2 mg/mL rat tail collagen I per well as per the manufacturer's protocol (Thermo Fisher Scientific, A10483-01). The collagen I seeded MRC-5 lung fibroblasts was then allowed to polymerize for 1 h in a humidified incubator at 37°C with 5% CO_2_. After gelation, 200,000 A549 cells in 2 mL DMEM 10% FBS were added on top of MRC-5-embedded collagen-I gels and incubated for a further 24 h to enable a confluent layer to form.

To establish 3D alveolar organoid models, a 24-well plate was first coated with matrix solution made up of 40% Matrigel (Corning Life Sciences, Corning, NY) and 60% DMEM/10% FBS (Thermo Fisher Scientific) polymerized at 37°C for 1 h. A 300,000 A549 and 30,000 MRC-5 cell mixture was prepared in a 5% Matrigel/DMEM (10% FBS) solution and added onto the solidified Matrigel layer per well. This enabled the development of alveolar organoids after culturing in standard conditions for 21 days, with media replenishment every 2 days. Organoid development was monitored every day and recorded on days 3, 9, and 21 by imaging three regions of interest (ROIs) per well for analysis of organoid numbers, diameter, and area using Fiji (ImageJ, National Institutes of Health, Bethesda, MD).

On day 21, organoids were harvested whole using the Cultrex™ Organoid Harvesting Solution (Bio-Techne, Minneapolis, MN), as per the manufacturer's instructions. Harvested organoids were then embedded in 2 mg/mL rat tail collagen-I per well as per the manufacturer's protocol (Thermo Fisher Scientific), in 6-well Tissue Train circular foam plates (Flexcell International Corporation,). After collagen polymerization, organoids were cultured in DMEM 10% FBS (Thermo Fisher Scientific) for 24 h, before the media was changed to DMEM 1% FBS (Thermo Fisher Scientific) for strain experiments. 3D alveolar co-cultures and organoid non-strained control Tissue Train plates were maintained under static conditions, while the experimental plates were subjected to equibiaxial strain in the Flexcell as described below.

### Strain application

**Figure 1:**
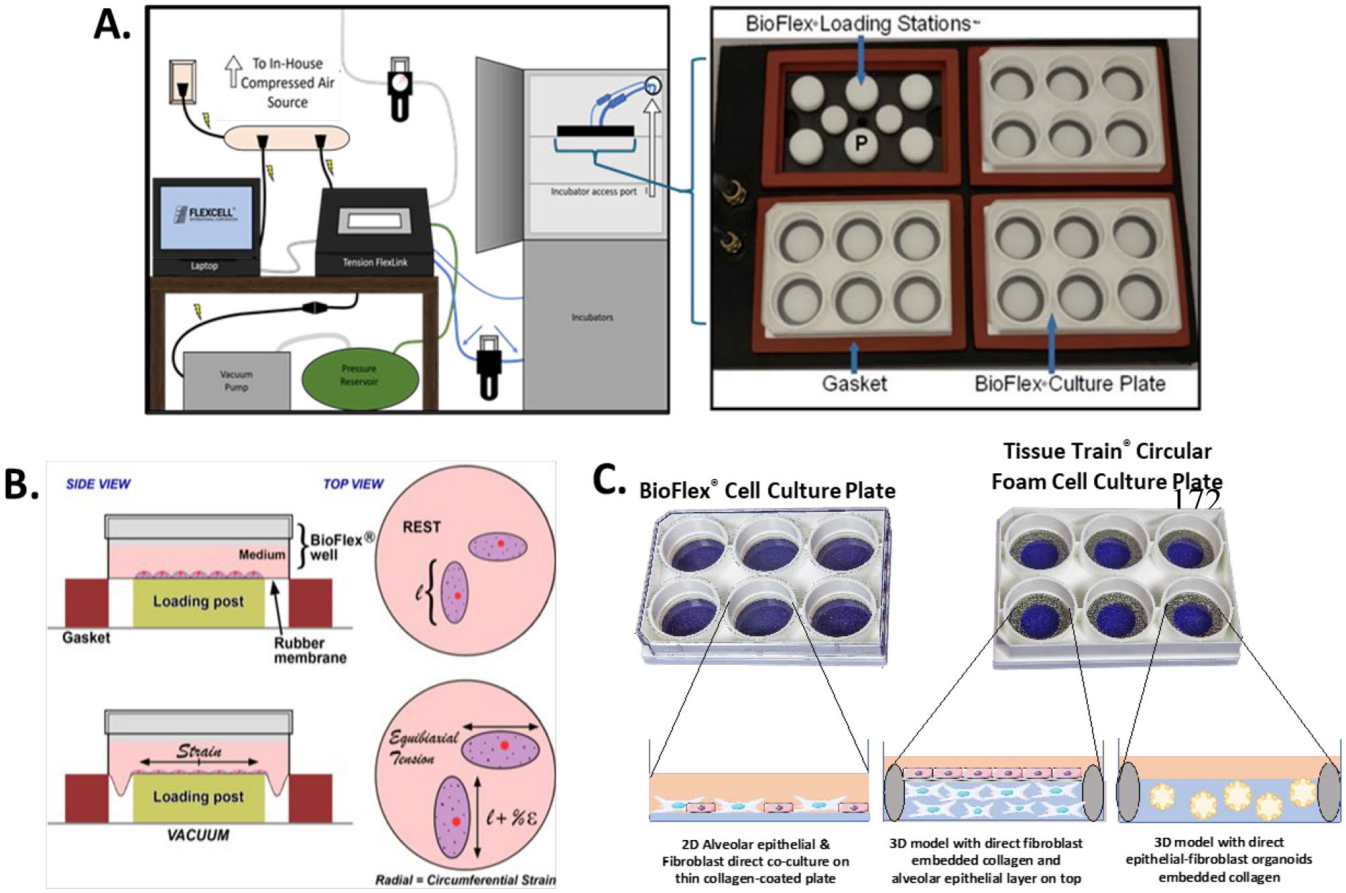
Overview of the Flexcell^®^ Tension system, mechanism of cell strain, and models used. **(A)** Schematic of the Flexcell^®^ FX-6000TM System, with components including: Surge protected power strip, power outlet, Ethernet cable, reinforced vacuum tubing, compressed air regulator/filter, vent tubing, system tubing, flex in tubing, flex out tubing, water trap, baseplate. To the right, enlargement of a top view of the baseplate in which the different culture plates sit for strain experiments showing white loading posts. **(B)** Schematic of top and side-view of membrane deformation and subsequent equibiaxial strain onto cells. **(C)** 2D alveolar epithelial-fibroblast co-cultures were seeded on BioFlex plates, whereas 3D co-cultures and epithelial-fibroblast organoids were seeded on the Tissue Train plate. Adapted from Flexcell^®^ International Corporation with written permission: USER MANUAL FLEXCELL^®^ DYNAMIC CULTURE SYSTEM FX-6000™ Tension 03-19-18 Rev 1.2, https://cdn.prod.website-files.com/5b916a469721fd4a8ae38199/5bb51aa931f6192010078b79_FX-6000TensionUsersManual.pdf, COPYRIGHT © 2018 FLEXCELL^®^ INTERNATIONAL CORPORATION, www.flexcellint.com; FLEXCELL Loading Station Tech Report, Rev 7.0 07-25-17, https://cdn.prod.website-files.com/5b916a469721fd4a8ae38199/5bb519b8e56d4d5aedf7e3c8_101_LoadingStationsTech.pdf, COPYRIGHT © 2009 FLEXCELL^®^ INTERNATIONAL CORPORATION, www.flexcellint.com.

Figure 1 illustrates the Flexcell system setup (A), membrane deformation process (B), and detailed descriptions of the models used in this study (C). 2D and 3D co-cultures as well as organoids in collagen I-coated 6-well BioFlex and 6-well Tissue Train circular foam culture plates were exposed to either pathological cyclic strain in the Flexcell FX-6000 Tension system (Flexcell International Corporation) or left as static non-strained controls. The regimen selected to mimic pathological conditions was an equibiaxial strain of 18% amplitude at a frequency of 0.4 Hz for a period of 24 h. Both BioFlex and Tissue Train plates have a flexible silicone elastomer membrane that is molded around a circular 25 mm static post by vacuum pressure during the strain regimen. After 24 h of strain application, the supernatant was collected from both the 2D models on BioFlex plates and 3D hydrogels on the Tissue Train circular foam plates for subsequent assays.

### Immunofluorescence analysis

After experiments, the 2D and 3D models were washed with Dulbecco's phosphate buffered saline (DPBS), fixed with 4% paraformaldehyde for 1 h at 4°C, washed again, and permeabilized with 0.2% Tween 20 at 4°C. Following another wash, the cultures were blocked with 5% bovine serum albumin for 1-h at room temperature. Cultures were incubated overnight at 4°C with a staining cocktail containing anti-zonula cccludens-1 (ZO-1) antibody conjugated with Alexa Fluor™ 594 (1:100; 339194, Invitrogen), Phalloidin Alexa Fluor™ 488 (A12379, 1:1,000; Thermo Fisher Scientific), and DAPI (D1306, 1:4,000; Thermo Fisher Scientific). Imaging was performed using a Leica DMi8 confocal microscope (Leica Microsystems, Wetzlar, Germany) for fluorescence intensity analysis (40X objective) and an EVOS M500 fluorescence microscope (Thermo Fisher Scientific) for morphology analysis (10X objective). For 2D and 3D hydrogel models, single images were captured from side and center fields per well, while for 3D organoid models, z-stacks at 5 μm thickness were acquired to capture their full depth, all representative images were taken at the same Z-plane, and imaging parameters—including laser power, gain, exposure time, and detector settings—were kept constant. Fluorescence intensity of the organoids was acquired using Z-stack imaging and quantified from the merged projection. Fluorescence intensity was analyzed using Fiji and corrected per cell for 2D models and per organoid and area for 3D models. Unstained internal controls were used for every imaging session. Global cell and organoid numbers were determined by point counts using two randomly selected fields of view per model, three models per experiment using automated image analysis. Morphological analysis also involved manual classification of MRC-5 cells as spindle or broadened and A549 cells as round or spindle, based on two fields of view per model, three models per experiment.

### Interleukin-6 (IL-6) and interleukin-8 (IL-8) ELISA and cellular death assay

To evaluate the release of inflammatory mediators in various experimental conditions, enzyme-linked immunosorbent assays (ELISA) were performed to measure IL-6 and IL-8 concentrations in the supernatants collected from both co-culture (and monoculture) models, under strained and non-strained conditions. IL-6 (D6050) and IL-8 (DY208) ELISA kits (R&D Systems, Minnesota, Minneapolis) were used following the manufacturer's protocols, and results were quantified using the BioTek Cytation 7 (Agilent, Santa Clara, California).

To assess cell cytotoxicity, the CyQUANT™ lactate dehydrogenase (LDH) Cytotoxicity Assay (C20300, Thermo Fisher Scientific) was performed according to the manufacturer's instructions. Supernatants from the experimental models were subjected to the LDH absorbance assay to quantify LDH released into the medium, as a measure of potential cell lysis and death in both strained and static models. LDH concentration was quantified using recombinant human LDH (Abcam, ab93699) by generating a standard curve at approximately half-log concentrations from 1 pg/mL to 5 ng/mL.

### Statistical analysis

Results are shown as the standard error of the mean of four or more independent experiments. Prism 10 (GraphPad, California, USA) was used as the statistical analysis software. The differences between paired observations were assessed with paired *t*-test while multiple comparisons are assessed with a 1 way and 2-way ANOVA with *post-hoc* Tukey test.

## Results

### Characterization of 2D co-culture morphology, tight junction marker, cytoskeletal changes, inflammatory markers, and marker of cell death

**Figure 2:**
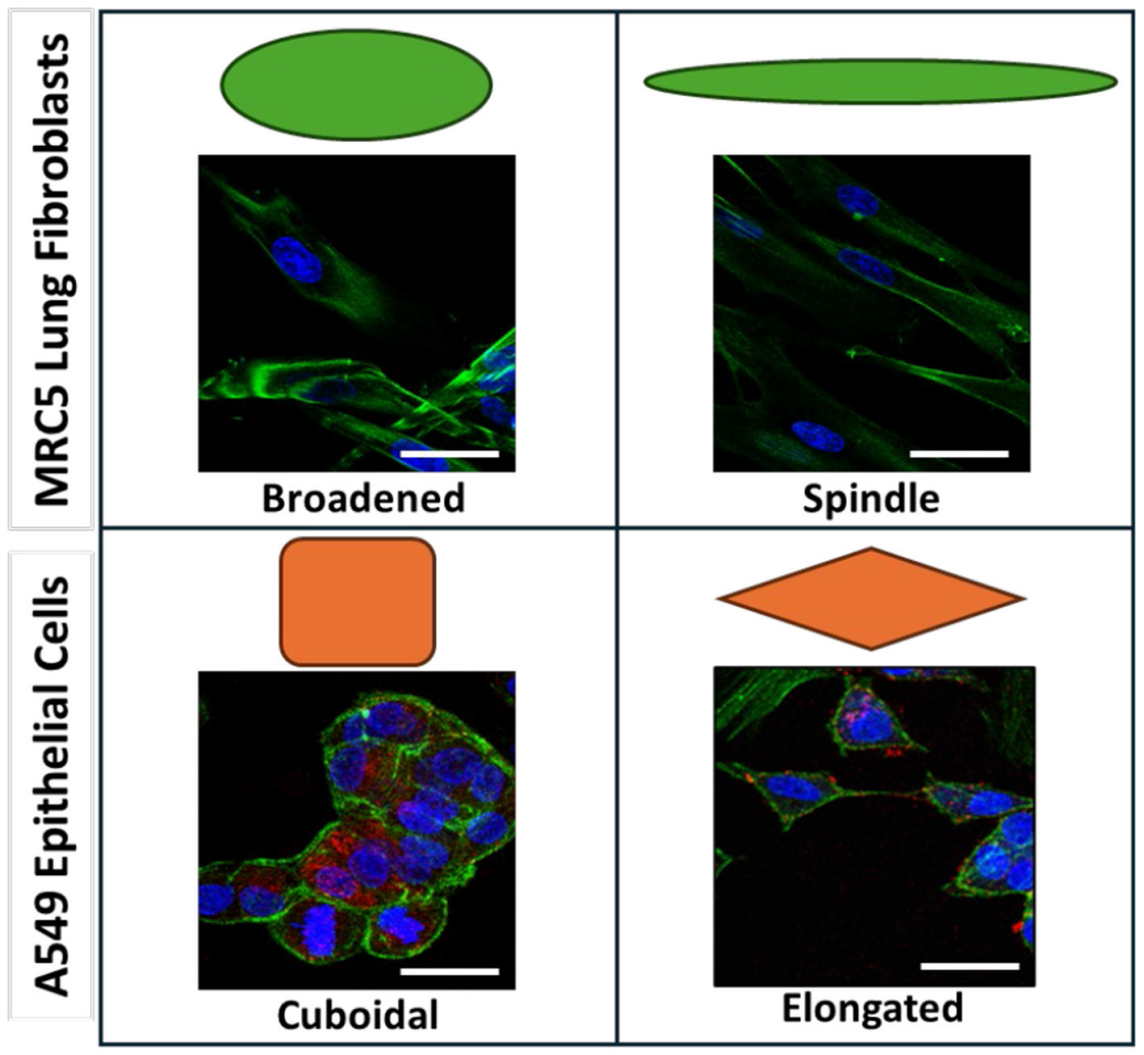
Representative immunofluorescence images of MRC-5 fibroblasts (stained with phalloidin) and A549 epithelial cells (stained with phalloidin and against ZO-1) showing distinct morphologies. Spindle-shaped cells are elongated, narrow, and fusiform with tapering ends, while broadened cells appear enlarged and flattened. In A549 cells, cuboidal morphology denotes a height roughly equal to width, whereas elongated cells are thinner and stretched. Cartoons of these morphologies are provided above each representative image. Scale bars represent 25 μm.

**Figure 3:**
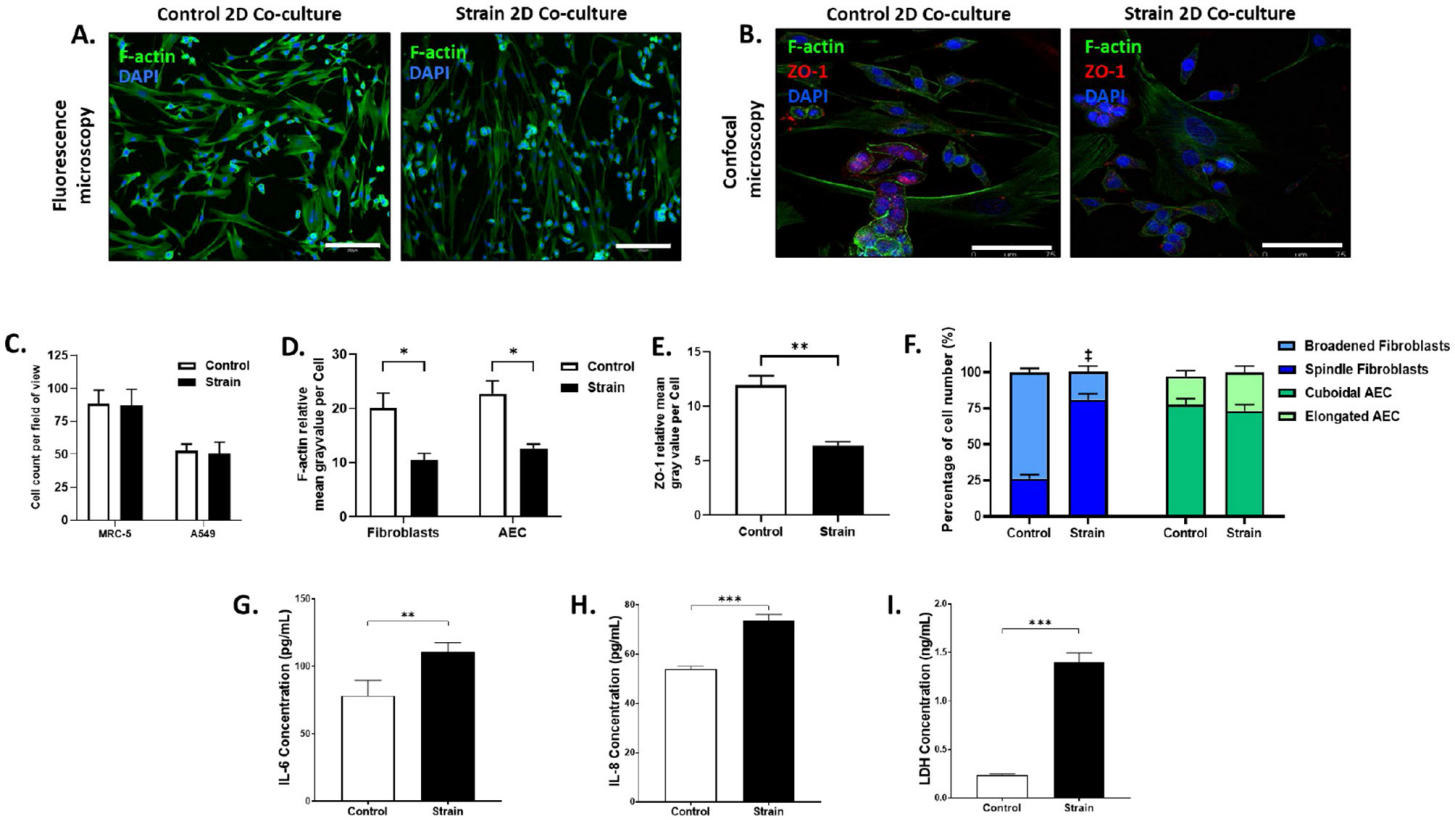
Exposure to pathologic strain regimen on morphology, tight junction marker, cytoskeletal changes, inflammatory markers, and marker of cell death in MRC-5 and A549 *2D* co-cultures. Co-cultures were subjected to pathologic strain regimen of equibiaxial strain at 18% amplitude and 0.4 Hz for 24 hours. **(A)** Representative fluorescence microscopy images at 10X objective stained for nuclei (blue) and F-actin (**green**). Scale bar represents 250 μm. **(B)** Representative confocal images of control and strained co-cultures stained for nuclei (**blue**), F-actin (**green**) and ZO-1 (**red**). Scale bar represents 75 μm. **(C)** Total number of fibroblasts and epithelial cells per field of view were counted and summarized. Cell counts are representative of each field of view. **(D)** Mean F-actin fluorescence intensities of fibroblasts and AEC are summarized. **(E)** Mean ZO-1 fluorescence intensity of AEC is summarized. **(F)** Proportions of cells exhibiting spindle or broadened morphology for fibroblasts and cuboidal or elongated morphology for AEC are summarized. ^**‡**^Control vs. strain, spindle (*p* = 0.0006) and broadened (*p* = 0.0005) **(G)** Interleukin-6 concentrations in the supernatant was assessed via ELISA. **(H)** Interleukin-8 concentrations in the supernatant were assessed via ELISA. **(I)** Lactate dehydrogenase, a marker for cell death, was quantified in the supernatant in strain and control conditions. Data reported is mean ± SEM indicated for 6 replicates, only pairwise comparisons *p* < 0.05 are included, *p < 0.05, **p < 0.01, ***p < 0.001.

2D A549 alveolar epithelial cell (AEC) and MRC5 lung fibroblast co-cultures were generated and exposed to cyclic strain. The resultant cultures were immunostained with phalloidin for cytoskeletal F-actin (essential protein for cell shape and motility) to count cells. After fluorescence imaging and cell counting, immunostaining for the junctional protein ZO-1 was performed to assess the effects of strain on cytoskeletal protein and junctional complex expression in 2D alveolar co-culture models. ZO-1 expression was more prominent in AEC than in fibroblasts (Figure 2) and was used to distinguish alveolar epithelial cells from lung fibroblasts, enabling cell morphology assessments. Based on preliminary experiments and previous literature, lung fibroblasts were categorized as spindle-shaped or broadened, while alveolar epithelial cells were classified as cuboidal or elongated as shown in Figure 2 (38, 39). Representative fluorescence images of non-strained static controls and strained 2D co-cultures are shown in Figure 3A. Figure 3B shows representative confocal images of non-strained static controls and strained AEC and lung fibroblast co-cultures stained for nuclei, F-actin, and ZO-1 to differentiate the two cell populations. ZO-1 expression was analyzed together with cytoskeletal F-actin to assess the effect of strain on junctional complex and cytoskeletal protein expression in 2D alveolar co-culture models. Here, we observed that cell numbers of A549 or MRC-5 were not significantly different between control and strained models (Figure 3C). F-actin intensity was however, significantly decreased in AECs and fibroblasts in the co-cultures after strain compared to static non-strained controls (Figure 3D). ZO-1 intensity in AECs significantly decreased with strain in both co-cultures and monocultures (Figure 3E and Supplementary Figure 2C). However, the reduction was significantly lower in co-cultures (*p* = 0.01). As ZO-1 staining enabled the identification of the alveolar epithelium, F-actin staining could be analyzed for the different cell-types. In monocultures, F-actin intensity significantly decreased with strain in both fibroblasts and AECs (Supplementary Figure 1B). Following strain, F-actin intensity in fibroblasts was comparable between monocultures and co-cultures. However, in AECs, F-actin intensity was significantly higher in co-cultures (*p* = 0.0006) after strain.

Next, the morphology of A549 and MRC-5 cells was assessed using cytoskeletal F-actin and ZO-1 staining. Representative images showed MRC-5 spindle-shaped cells are elongated, narrow, and fusiform with tapering ends, while broadened cells appear enlarged and flattened. In A549 cells, cuboidal morphology denotes a height roughly equal to width, whereas elongated cells are thinner and stretched (Figure 3F). When morphology was quantified, a significantly greater proportion of fibroblasts exhibited spindle morphology under strain compared to non-strained static controls (81.0 ± 4.0 % vs. 26.2 ± 2.7%), with the remaining adopting a broadened shape (Figure 3F). In contrast, AECs maintained their cuboidal shapes under both strain and control conditions with no significant differences observed (Figure 3F). Similar trends were observed in AEC and fibroblast monocultures. Under strain, 85.7 ± 1.4% of fibroblasts displayed spindle morphology, which was significantly greater than in static controls (18.0 ± 1.8%) and comparable to fibroblast morphology proportions in 2D co-cultures (Supplementary Figure 1A). In monocultures, AEC morphology showed no significant differences between strain and control (Supplementary Figure 1B).

The effect of mechanical strain on the release of inflammatory mediators and cell death in 2D alveolar co-cultures were characterized by measuring the release of IL-6, IL-8 and lactate dehydrogenase (LDH) in the supernatant of strained and control 2D co-cultures. Here, it was found that 18%, 0.4Hz strain for 24 h caused a significantly increased release of IL-6 and IL-8 in 2D co-cultures compared to control (Figures 3G, H). There was also a significantly increased release of LDH after strain of 2D co-cultures compared to controls (Figure 3I). IL-6 release from fibroblast monocultures increased significantly under strain (Supplementary Figure 2C), while IL-8 release was significantly elevated in both fibroblast and AEC monocultures under strain (Supplementary Figure 2D). LDH was also significantly higher with strain in both fibroblast and AEC monocultures, but the LDH release between fibroblasts and AEC with strain was not significantly different (Supplementary Figure 2E).

### Characterization of 3D co-culture morphology, tight junction marker, cytoskeletal changes, inflammatory markers, and marker of cell death

**Figure 4:**
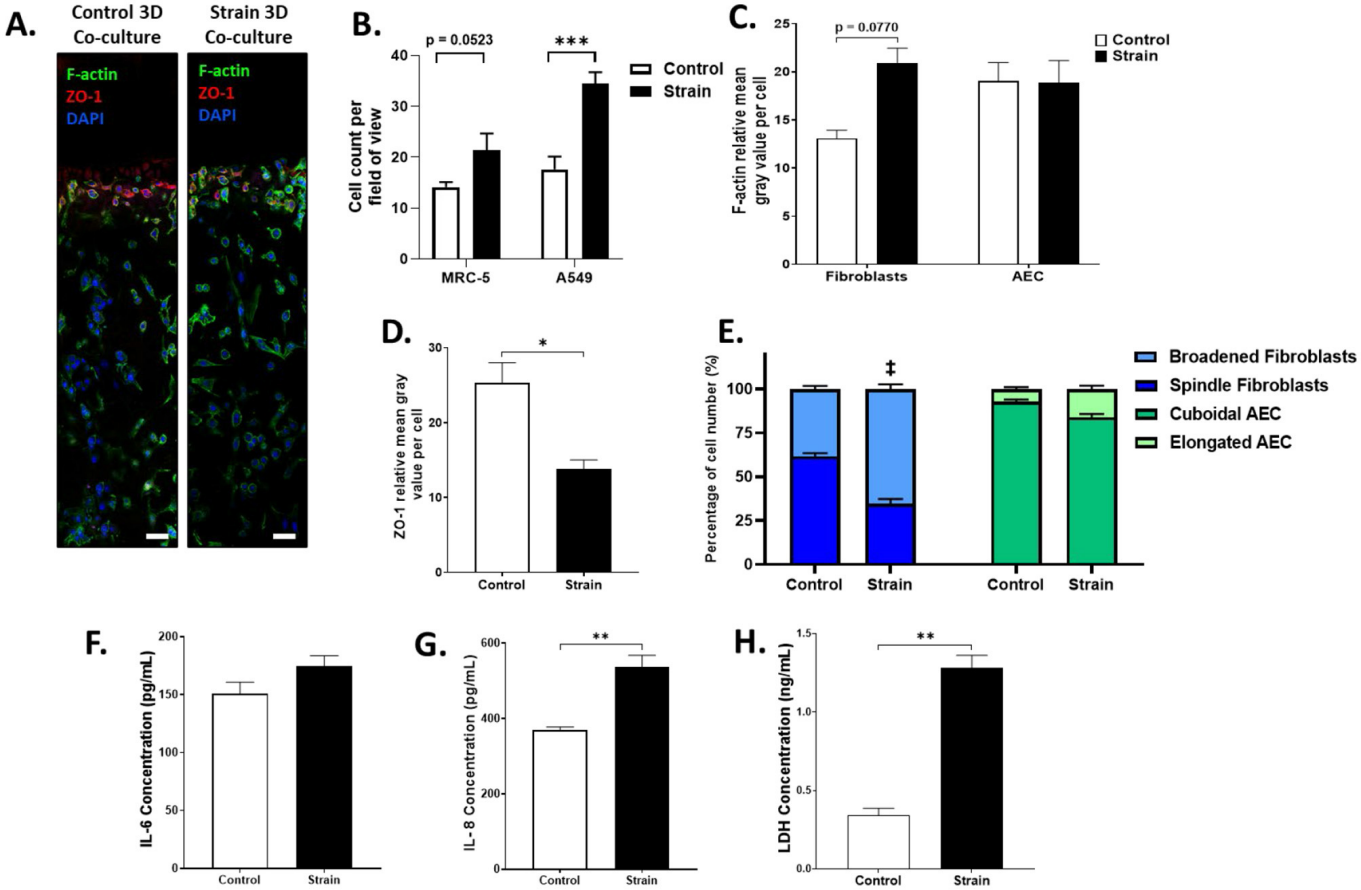
Exposure to pathologic strain regimen on morphology, tight junction marker, cytoskeletal changes, inflammatory markers, and viability MRC-5 and A549 *3D* co-cultures. Co-cultures were subjected to the pathologic strain regimen for 24 h. **(A)** Representative confocal images of control and strained co-cultures stained for nuclei (**blue**), F-actin (**green**) and ZO-1 (**red**). Scale bar represents 75 μm. **(B)** Total number of fibroblasts and epithelial cells per field of view were manually counted and summarized. Cell counts are representative of each field of view. **(C)** Mean F-actin fluorescence intensities of fibroblasts and AEC are summarized. **(D)** Mean ZO-1 fluorescence intensity of AEC was summarized. **(E)** Proportions of cells exhibiting spindle or broadened morphology for fibroblasts and cuboidal or elongated morphology for AEC are summarized. MRC-5 and A549 cells were distinguished by differences in F-actin and ZO-1 expression as well as cellular morphology. ^**‡**^Control vs. strain, spindle (*p* = 0.05) and broadened (*p* = 0.05) **(F)** IL-6 concentrations in the supernatant were assessed via ELISA. **(G)** IL-8 concentrations in the supernatant were assessed via ELISA. **(H)** LDH was quantified in the supernatant in strain and control conditions. Data reported is mean ± SEM indicated for 6 replicates, only pairwise comparisons *p* < 0.05 are included, *p < 0.05, **p < 0.01, ***p < 0.001.

Representative cross-sectional confocal images of the control and strained 3D alveolar epithelial-fibroblast co-culture hydrogels stained for F-actin, ZO-1, and cell nuclei are shown in Figure 4A. After the application of a 24 h 18%, 0.4 Hz strain, a significant increase in A549 number was observed whereas MRC-5 cell numbers were not significantly different (Figure 4B). F-actin intensity was not significantly different compared to static non-strained controls for both lung fibroblasts and AECs (Figure 4C). However, 18%, 0.4 Hz strain significantly reduced ZO-1 expression by 45.2% in AECs compared to non-strained static controls (Figure 4D). A significant decrease in the proportion of fibroblasts exhibiting spindle morphology compared to control (34.6 ± 2.8% vs. 61.6 ± 1.8%) was observed (Figure 4C). The proportion of AECs with cuboidal morphology did not significantly change with strain (Figure 4C). Strain conditions did not significantly affect supernatant IL-6 concentrations; however, it significantly increased IL-8 concentrations (Figures 4F, G) compared to controls. Added to this, LDH release was significantly increased after strain application in 3D alveolar co-cultures compared to controls (Figure 4H).

### Characterization of alveolar epithelial-fibroblast organoid growth and maturation, model morphology, tight junction marker, and cytoskeletal changes

**Figure 5:**
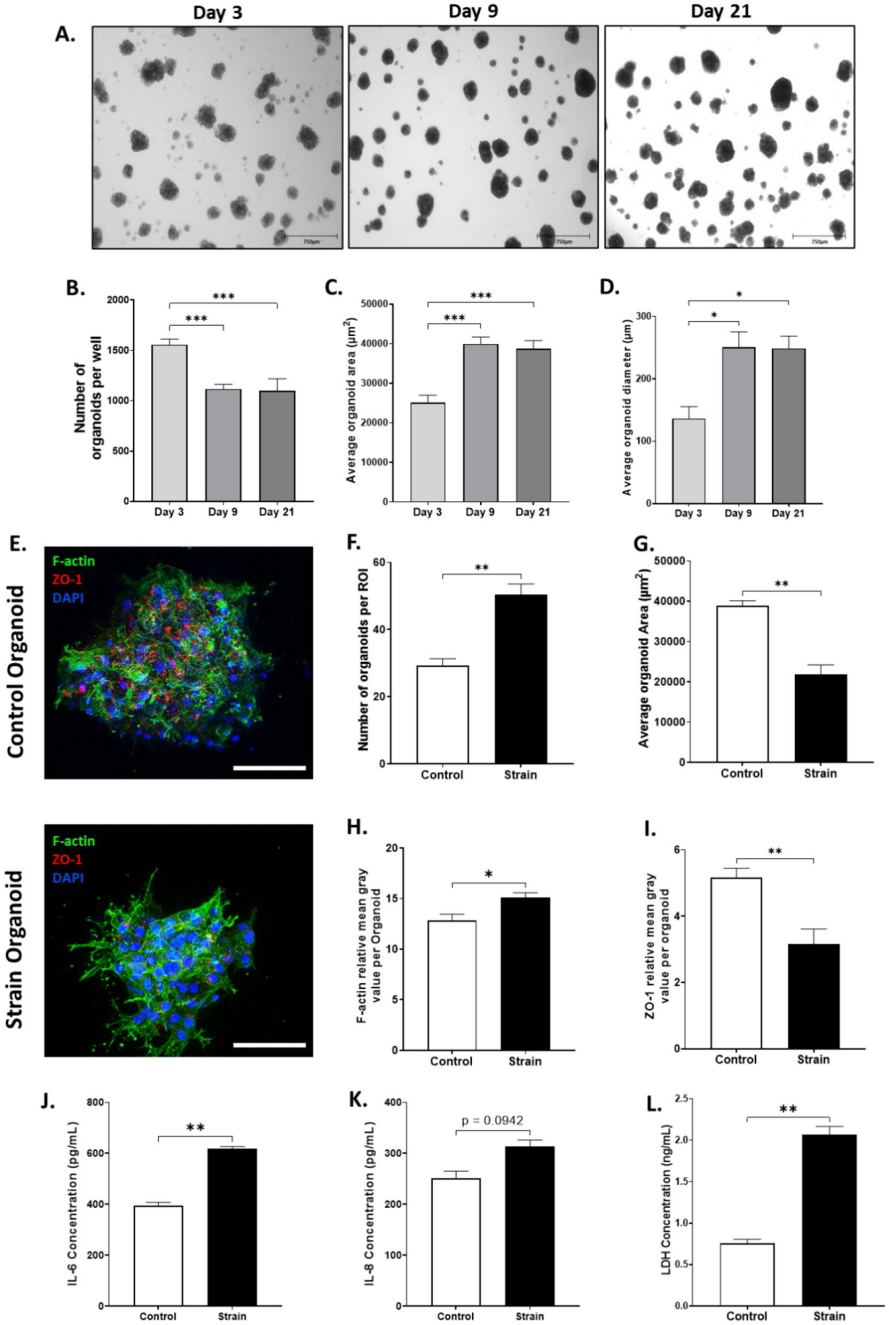
Evaluating development before strain and morphology, tight junction marker, cytoskeletal changes, inflammatory markers, and cell death after strain of alveolar epithelial-fibroblast organoid models. **(A)** Representative brightfield microscopy image of the organoid development in the hydrogel at 4× objective in days 3, 9, and 21. Scale bar = 350 μm. Parameters of organoids that were measured were **(B)** Number of organoids per well, **(C)** Average organoid area, and **(D)** Average organoid diameter (only organoids over 50 μm diameter were included). Organoid development data reported as mean ± SEM indicated for 4 technical replicates, *n* = 4. *p < 0.05, ***p < 0.001 **(E)** Representative confocal microscopy images at 40× objective stained for nuclei (**blue**), F-actin (**green**), and ZO-1 (**red**). Scale bar represents 75 μm. **(F)** Number of organoids were counted per region of interest. **(G)** Average area of organoids was assessed and summarized. **(H)** Mean F-actin fluorescence intensities of the organoids are summarized. **(I)** Mean ZO-1 fluorescence intensity of the organoids was summarized. **(J)** IL-6 concentrations in the supernatant were assessed via ELISA. (**K)** IL-8 concentrations in the supernatant were assessed via ELISA. **(L)** LDH was quantified in the supernatant in strain and control conditions. Characterization after strain data reported is mean ± SEM indicated for 4 replicates, *p < 0.05, **p < 0.01, ***p < 0.001.

Alveolar epithelial-fibroblast organoids were established and cultured over 21 days, with organoid numbers, area, and diameter assessed at days 3, 9, and 21 to monitor growth and development. Representative brightfield microscopy images show organoids exhibited a circular morphology as expected throughout the maturation period (Figure 5A). The average number of organoids per well was significantly lower at days 9 and 21 compared to day 3 (Figure 5B). Complimentarily, the average organoid area and diameter was significantly higher at days 9 and 21 compared to day 3 (Figures 5C, D). The averages of these three measurements are reported in Supplementary Table 1. From these indices, 24 000 organoids were consistently produced, embedded in collagen-I-hydrogels and subjected to the 24 h 18%, 0.4 Hz strain.

Figure 4E shows representative confocal images of non-strained static control and strained alveolar epithelial-fibroblast organoid models immunostained for F-actin, ZO-1, and cell-nuclei. After the application of a 24 h 18% 0.4 Hz strain the number of organoids significantly increased by 42%, whereas organoid area decreased by 43% compared to control (Figures 5F, G). It was then observed that lung fibroblasts developed F-actin positive cytoplasmic projections that distorted the spherical shape of organoids upon strain. In line with this, there was a significant increase in F-actin fluorescence intensity in organoids following the application of strain compared to static controls (Figure 5H). Plotting F-actin intensity as a function of Z-distance in organoid Z-stacks revealed elevated F-actin levels near the center of strained organoids, with comparable expression at the peripheries (Supplementary Figure 3A). Further, ZO-1 fluorescence intensity was significantly lower in AECs after strain (Figure 5I). Compared to controls, ZO-1 expression was consistently reduced at all Z-distances in strained organoids (Supplementary Figure 3B). Secreted IL-6 concentrations were significantly increased with strain in line with an increased, non-significant release of secreted IL-8 (Figures 5J, K). As found in the previous models, LDH was also significantly increased in strained organoids compared to static controls (Figure 5L).

## Discussion

In this brief report, we developed and characterized mechanically strained 2D and 3D multicellular alveolar epithelial-fibroblast co-culture and organoid models using the Flexcell system. After models were characterized, it was determined that the application of a 24-h pathophysiological equibiaxial strain of 18% amplitude, 0.4 Hz, caused significant changes in the proliferation, of 3D alveolar epithelial-fibroblast co-culture and organoid models, but not in 2D co-cultures. There were also significant reductions in F-actin levels in both AECs and lung fibroblasts in 2D models, as opposed to increased F-actin expression in 3D models due to strain. We also observed a significant increase in A549 cell numbers with strain in 3D co-cultures. All models showed a decrease in the epithelial tight junctional protein ZO-1 in response to strain. Furthermore, there was increased pro-inflammatory cytokine release (IL-6, IL-8) and decreased viability in all multicellular models due to pathophysiological strain.

Various biomimetic models have been established ranging from simple 2D (transwell) co-cultures to conditioned medium exposure models, to 3D hydrogel embedded cellular models, organoids, and microfluidic lung-on-chip models to mimic the lung's microenvironment (11). Mechanical bioreactors such as the Flexcell have gained popularity over the years and are able to introduce the element of the mechanical environment in these models which was hither-to be understudied (11). In the present study, we combined 3D multicellular epithelial-fibroblast models and the Flexcell bioreactor to mimic the effects of strain in the alveolar in the multicellular lung environment. Lower strain amplitudes, (5–10%) are used to mimic normal lung expansion during quiet tidal breathing (3, 7) whereas higher strain amplitudes (15–20% in 3D models and up to 30% in 2D models) are associated with pathophysiological conditions such as mechanical injury and tissue deformation in lung diseases (32, 33, 40–42). In this study, we observed that an 18%, 0.4 Hz equibiaxial strain mimicked pathophysiological dynamic mechanical conditions to alter the structure and function of AECs and lung fibroblasts. Future studies would explore the use of the complex models established here to assess and compare more physiological strain conditions.

Cellular proliferation, plays a critical role in lung tissue homeostasis and repair, particularly under mechanical forces associated with breathing (43). While no significant changes in proliferation were observed in the 2D alveolar co-culture model with pathological strain, total cell and organoid numbers were significantly higher in 3D co-cultures and organoids. This aligns with literature demonstrating that 3D matrix environments promote strain-induced proliferation, a response driven in part by epithelial-mesenchymal crosstalk, whereas 2D monolayers are often limited in their ability to replicate these interactions (19, 44). Mechanical strain can activate pathways that enhance cell proliferation; however, when applied cyclically, the repetitive deformation may lead to distinct cellular responses, such as increased expression of mesenchymal markers, particularly in compliant matrices (45). However, it is important to note that the proliferative response to cyclic mechanical strain, such as in fibroblasts, can depend on factors like strain duration and collagen scaffold stiffness (46, 47), and these context-dependent variables must be considered when interpreting results across different culture systems. It is important to note that as this study is a brief report, a more comprehensive assessment of proliferation with markers such as Ki67 and incorporation of nucleotide analogs in replicating cells (48) should be done in the established multicellular mechanical models for future studies. These results highlight the ability of the 3D co-cultures and organoids to better mimic the mechanical *in vivo* lung environment compared to 2D models. This study's 3D alveolar-fibroblast models provide a powerful platform for investigating how mechanical forces and multicellular interactions contribute to lung diseases which are characterized by impaired repair processes, altered epithelial-fibroblast crosstalk, and excessive ECM deposition (49–51).

To further characterize established mechanical multicellular models, the effects of pathological strain on cellular morphology, cytoskeletal structure, and tight junction protein expression were determined. Changes in the cellular morphology of MRC-5 and A549 cells were assessed through immunofluorescent staining, with ZO-1 expression (an epithelial cell-specific marker) and distinct cell morphology serving as key differentiators between the two cell types. The shapes adopted by fibroblasts and AECs have been previously observed and are influenced by factors such as mechanical strain, extracellular environment changes, and their lung region of origin (38, 39, 52–54). Here, in either 2D or 3D models, AECs displayed no significant morphological changes in response to mechanical strain, suggesting that the specific strain conditions used may not regulate this aspect of their phenotype. However, ZO-1 a tight junctional protein important for epithelial barrier function, was significantly reduced after the application of pathological strain in all multicellular models suggesting a strong effect of strain on epithelial barrier dysfunction. ZO-1 expression was also consistently decreased across all Z-distances in organoids, suggesting a lack of Z-position-dependent variation. Possible mechanisms underlying this may include mechanical disruption of the actin cytoskeleton and decreases in intracellular ATP levels which are critical for maintaining junctional integrity (55). Consequences of barrier disruption can include increased alveolar permeability, inflammation, fibrosis, and increased susceptibility to infections (56, 57). To further explore these findings, assessments such as paracellular permeability assays, transepithelial electrical resistance and electrical cell impedance sensing are necessary to evaluate functional changes in barrier integrity (57, 58).

The lung fibroblasts used in our multicellular mechanical models exhibited a notable morphological shift to a spindle shape in 2D co-cultures after pathophysiological strain, while in 3D co-cultures, lung fibroblasts maintained a broadened myofibroblast-like morphology. This broadened shape, has been linked to fibroblast-to-myofibroblast differentiation, which points the potential for pathological strain to promote lung fibroblast activation, shown to drive ECM production and tissue remodeling and linked in separate studies to lung mechanical activation (11, 38). To further elucidate this activation process, future studies should assess the development of contractile actin-myosin bundles and the expression of α-smooth muscle actin, which are hallmark features of myofibroblast activation (59, 60). Cytoskeletal F-actin showed a significant reduction in intensity in 2D fibroblast monocultures under strain while no significant changes were observed in F-actin of fibroblasts in the 2D alveolar epithelial-fibroblast co-cultures and 3D co-cultures. However, F-actin expression was elevated in strained epithelial-fibroblast organoids, with the highest levels observed near the organoid center. Although the underlying mechanisms require further investigation, this suggests that AECs and their associated crosstalk may play a compensatory role in mitigating cytoskeletal changes in response to mechanical strain. For instance, mechanical stimulation may enable AECs to signal to fibroblasts via the prostaglandin E2 pathway, potentially counteracting strain-induced alterations (61, 62).

While immune cells are traditionally seen as primary contributors to disease through the production of pro-inflammatory cytokines, it is now known that structural cells, such as AECs and fibroblasts also play crucial roles in driving lung inflammation (63). In this study, we explored the strain-induced release of pro-inflammatory cytokines IL-6 and IL-8 in 2D and 3D alveolar epithelial-fibroblast co-cultures and organoids. IL-6 is pivotal in the acute-phase response, driving the production of proteins by the liver and promoting the differentiation of B cells into plasma cells (64, 65). IL-8, a key chemokine, primarily facilitates the recruitment and activation of neutrophils at sites of inflammation (66). It was found that exposure to pathological strain predominantly increased lung fibroblast derived IL-6 release while IL-8 was primarily produced by epithelial cells, corroborating published literature (67). In 3D co-cultures, strain-induced IL-8 release was observed, but IL-6 levels remained unchanged. Conversely, organoids exhibited increased release of both cytokines under strain. This indicates that the mechanical lung's 3D microenvironment potentially alters cytokine responses compared to monocultures, highlighting the importance of further investigating epithelial-fibroblast interactions in these more physiologically relevant models. In previous studies epithelial-fibroblast crosstalk has been linked to inflammatory release and potential miRNA regulation (50, 68). Future studies should investigate the mechanotranstruction pathways involved in driving IL-6, IL-8 and inflammatory cytokine release in the lung's complex mechanical multicellular microenvironment.

Cell death was assessed by measuring the release of LDH, a widely used marker for damage and membrane integrity (69, 70), in the strained mechanical co-culture models. Previous studies have shown that strain can compromise cell viability in AECs and lung fibroblasts, due to membrane damage and alterations in cytoskeletal dynamics (67, 71, 72). In this study, pathological strain increased LDH release in all models tested, with lung fibroblasts showing higher LDH sensitivity to strain than AECs. Notably, LDH release in 3D models was significantly higher—~50% higher than in 2D models. These heightened responses could be potentially attributed to the mechanical properties of the 3D model which mimics the ECM stiffness and spatial organization contributing to differential responses of AECs and lung fibroblasts. In addition, we observed an increase in cell numbers in 3D co-cultures concomitantly with an increase in LDH release. This could suggest strain induces sublethal or reversible membrane injury that releases LDH, but does not lead to apoptosis (73), localized areas of cytotoxicity which is not reflected with whole-field quantification techniques, or a possible compensatory proliferation effect in response to mechanical injury, which has been observed in other strain models (74). Future work to assess cell viability should use dyes/markers such as propidium iodide to specifically identify cells, use of apoptotic markers such as initiator or terminal caspase expression and mitochondrial integrity (75), together with assessment of mechanotransduction pathway factors involved in driving cell death responses in multicellular models.

This study has some limitations. (1) We used morphological analysis to distinguish between fibroblasts from epithelial cells and to observe their structural changes in response to strain. However, further studies are needed to assess corresponding functional changes, correlate them with structural alterations, and confirm findings using specific markers. (2) While the Flexcell system is a widely used platform for applying strain to cells, alternative systems such as the in-house device developed by Mondoñedo et al. (76) may offer improved performance. Their system demonstrated lower intra-well variance in area strain compared to the Flexcell BioFlex culture plates, particularly at higher strains. Thus, we cannot fully rule out the possibility of nonuniform deformation in our experiments, even though the Flexcell system is designed to apply equibiaxial strain. These findings underscore the importance of strain uniformity and suggest that exploring alternative stretching platforms may improve reproducibility across studies. (3) Our 3D model may introduce differential strain between the apical and basal regions due to thickness of the hydrogel and matrix softness. Prior studies have shown that in soft matrices like collagen-I that are membrane adherent, strain transmission decreases with depth, particularly near the unanchored apical surface (77). Although we did not directly measure intra-gel strain, *in situ* gel thickness, or characterize changes in the expression of relevant cell markers such as F-actin or ZO-1 with respect to Z-distance throughout the gel, we acknowledge this as a limitation of the study. Future work will aim to address this by using thinner constructs, modifying matrix stiffness, or incorporating embedded sensors to map strain distribution. (4) Our organoid model used A549 and MRC-5 cell lines, selected for their consistency, ease of use, and extensive characterization in the literature. However, these cell lines have some limitations. A549 cells are derived from lung adenocarcinoma and do not fully represent the phenotype of primary alveolar epithelial cells, especially in short-term culture (78). Similarly, MRC-5 cells, derived from fetal lung fibroblasts, may lack the heterogeneity and maturity of fibroblast populations in adult lung tissue. As a result, their responses to mechanical strain may not accurately reflect those of primary cells *in vivo*. (5) Lastly, the matrix in which the organoids are embedded does not fully recapitulate the native ECM rich in collagen, laminin, elastin, and other ECM proteins some of which are required for the specific function of alveolar epithelial cells and fibroblasts (30, 79, 80). Other models, such as precision cut lung sections, may also be explored due to their representation of the multicellular and native lung ECM (81).

In conclusion, we developed a multicellular alveolar model to evaluate how the mechanical microenvironment influences epithelial and fibroblast morphology. This model, combining easy-to-obtain basal alveolar epithelial and fibroblast immortalized cell lines with a collagen hydrogel and the Flexcell system, is easy to establish and adaptable for use in various labs to study mechanical strain. By assessing the structure and function of pathologically strained multicellular AEC- lung-fibroblast, 2D and 3D co-cultures, as well as organoids, we characterized distinct responses that serve as a basis for future studies in this brief research report. This study also underscores the utility of complex bioreactors such as the Flexcell tension system to mimic pathological breathing dynamics of various lung diseases via effects on cellular proliferation, morphological cellular, cytoskeletal, junctional protein, inflammatory and viability changes. These findings emphasize the impact of 3D co-culture and spatial organization on structure and function and the physiological relevance of 3D models in replicating the mechanical *in vivo* lung environment. These models therefore serve as a robust platform for studying mechanobiological responses in lung diseases involving fibrosis, ECM remodeling, and inflammation.

## Data Availability

The original contributions presented in the study are included in the article/Supplementary material, further inquiries can be directed to the corresponding author.
