# Supplementary material for: Dynamic mechanical stimulation of alveolar epithelial-fibroblast models using the Flexcell tension system to study of lung disease mechanisms
Source: Front Med (Lausanne). 2025 Aug 18;12:1552803. doi: 10.3389/fmed.2025.1552803 (PMC12401065; doi:10.3389/fmed.2025.1552803)
Supplement: Supplementary file 1 [file Supplementary_file_1.docx]

**Supplemental**


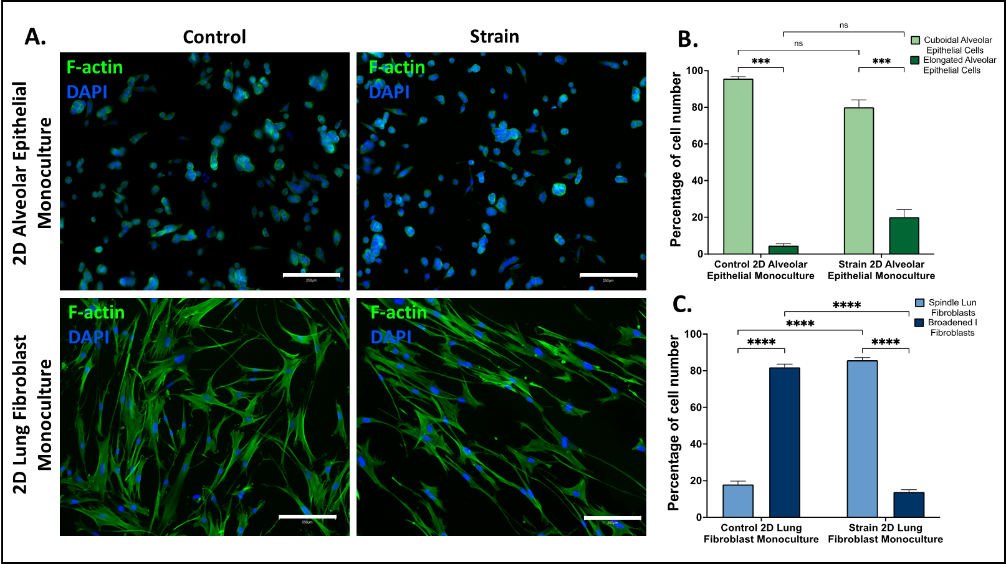


**Supplementary Figure 1. Morphology of MRC-5 and A549 2D monocultures subjected to pathological strain regimen. A.** Representative fluorescence microscope image at 10X magnification stained for nucleus (blue) and F-actin (green) in MRC-5 and A549 2D monocultures. **B.** Percentage of cell number of cuboidal and elongated alveolar epithelial cells in 2D alveolar epithelial monoculture model. **C.** Percentage of cell number of spindle and broadened lung fibroblasts in 2D lung fibroblast monoculture model. Data reported as mean ± SEM indicated for 6 replicates, n=6. *** p < 0.001, **** p < 0.0001


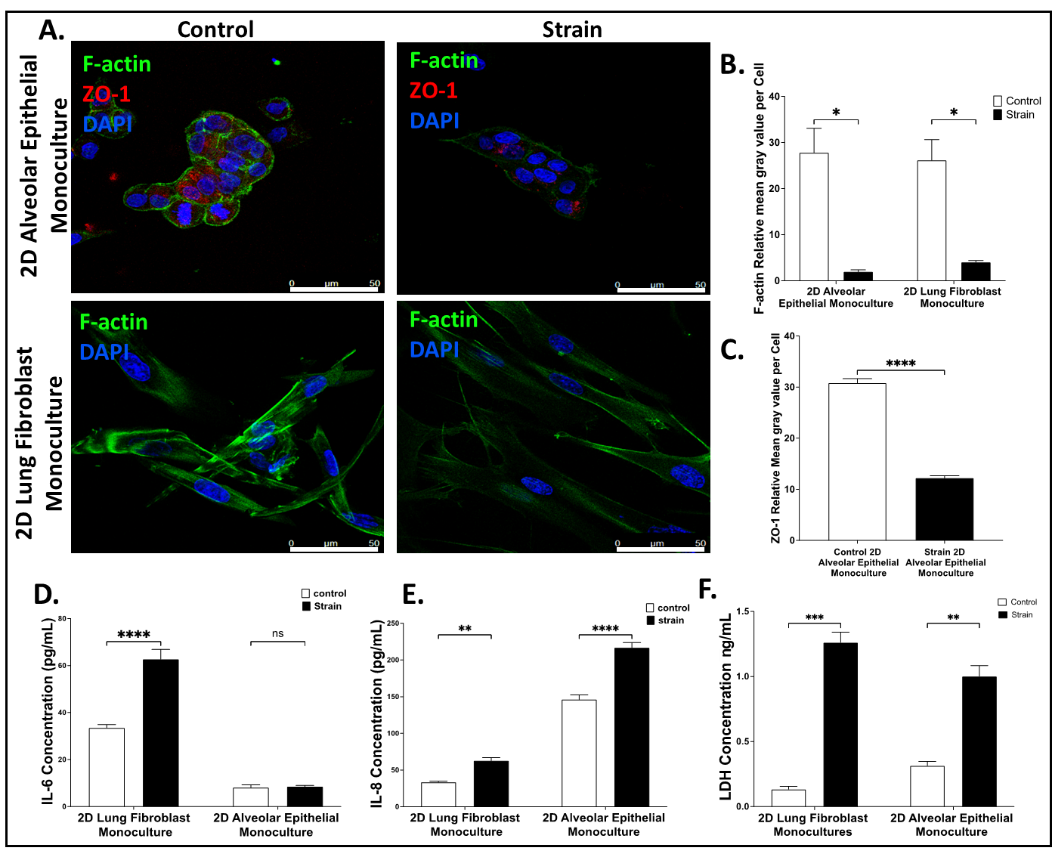


**Supplementary Figure 2. Characterization of cytoskeletal, tight junction, inflammatory and cell viability markers of MRC-5 and A549 2D monocultures subjected to pathological strain regimen. A.** Representative fluorescence microscope image at 40X magnification stained for nucleus (blue), F-actin (green), ZO-1 (red) in MRC-5 and A549 2D monocultures. **B.** Mean F-actin fluorescence intensity was quantified. **C.** ZO-1 fluorescence intensity in alveolar epithelial monocultures was quantified. **D.** Secreted IL-6 in MRC5 and A549 monocultures was quantified using ELISA. **E.** Secreted IL-8 from monocultures was quantified. **F.** LDH was quantified. Data reported as mean ± SEM indicated for 6 replicates, n=6. *** p < 0.001, **** p < 0.0001

**Supplementary Table 1. Growth and morphological measurements of alveolar epithelial-fibroblast organoids over a 21 day culture period**

|  | **Day 3** | **Day 9** | **Day 21** |
| --- | --- | --- | --- |
| **Mean number of organoids per well** | 1556±28 | 1117±22 | 1096±60 |
| **Mean organoid area (μm^2)^** | 2.51×10^4^±952 | 3.99×10^4^±868 | 3.87×10^4^±1040 |
| **Mean organoid diameter (μm)** | 137.6±9.5 | 250.6±12 | 248.9±9.9 |


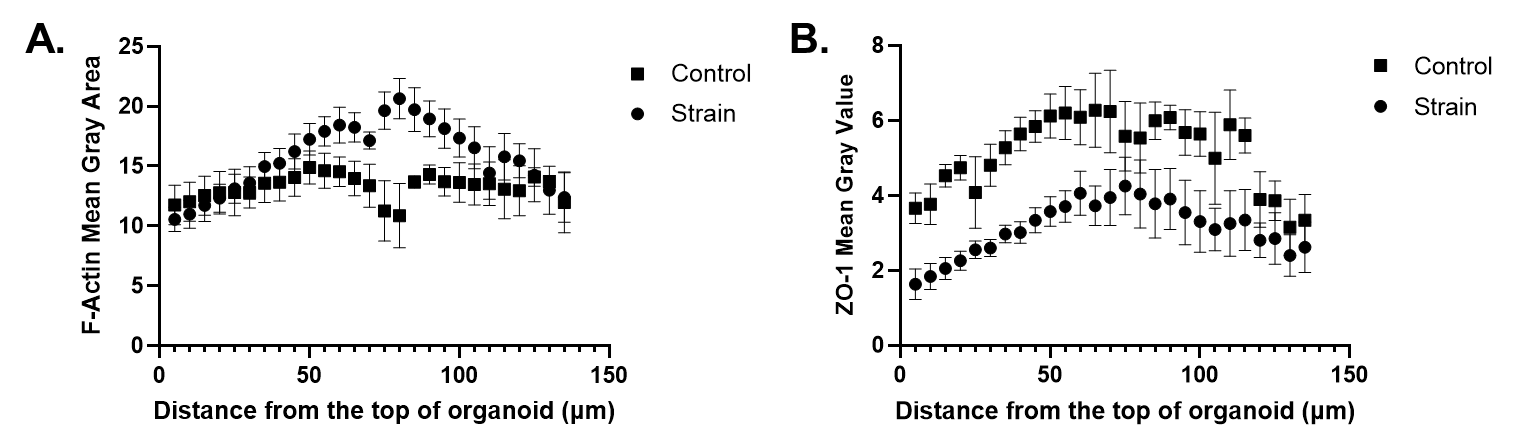


**Supplementary Figure 3. F-actin and ZO-1 expression quantified through Z-stacks of strained and control alveolar-fibroblast organoid models. A.** F-actin and **B.** ZO-1 fluorescence intensity (measured as mean gray value) were quantified at 5 μm intervals along the Z-stack, from the top to the bottom of the organoid, under both control and strained conditions. Four organoids were analyzed per condition.
